# Patients reasons for obtaining psychotropic medications without a prescription at retail pharmacies in Central Saudi Arabia

**DOI:** 10.17712/nsj.2016.4.20160245

**Published:** 2016-10

**Authors:** Fahad D. Alosaimi, Fay S. Alruwais, Fadah A. Alanazi, Ghaida A. Alabidi, Nadia A. Aljomah, Nuha S. Alsalameh

**Affiliations:** *From the Department of Psychiatry (Alosaimi), King Saud University Medical City, and from the College of Medicine (Alruwais, Alanazi, Alabidi, Aljomah, Alsalameh), King Saud University, Riyadh. Kingdom of Saudi Arabia*

## Abstract

**Objective:**

To explore the possible causes behind adults seeking psychotropic medications without a prescription; identify the most commonly used psychotropic medications without a prescription; and determine the prevalence of depression and anxiety disorders among adults who used psychotropic medications without a prescription in Riyadh, Kingdom of Saudi Arabia.

**Methods:**

A cross-sectional study was conducted from November 2014 to August 2015. A convenience sample was taken by distributing a self-administered questionnaire among participants who had obtained psychotropic medications without a prescription from retail pharmacies during the 4 weeks prior to study intake in Riyadh, Kingdom of Saudi Arabia. In addition to the study questionnaire, the 9-item Patient Health Questionnaire was used to screen for major depressive disorder, and the 7-item Generalized Anxiety Disorder Scale was used to screen for general anxiety disorders.

**Results:**

Of the 302 subjects, 42.4% attributed their use of psychotropic medications without a prescription to the non-seriousness of their symptoms and 28.5% to the high cost of psychiatric services. Escitalopram was the most commonly used medication (31.8%), and 3 atypical antipsychotics were used by more than one-fifth of the study participants. The prevalence for major depressive disorder was 46.0% and 41.7% for generalized anxiety disorder.

**Conclusions:**

Most of the participants were able to easily obtain psychotropic medications without a prescription. We recommend implementing strong policies to prevent retail pharmacies from dispensing psychotropic medications without a prescription.

Psychiatric disorders remained mostly untreated or improperly managed in the general population even in developed countries.^1^ Moreover, in a major USA study, 58% who were prescribed at least 1 psychotropic medication, had received no psychiatric diagnosis.^2^ In developing countries, people are commonly obtaining prescription-only medications from community pharmacies without seeking medical advice.^3^,^4^ In 2011, a study carried out in Jeddah, Saudi Arabia investigated dispensing behavior in retail pharmacies and reported that out of 38 requests, all pharmacists dispensed fluoxetine (an antidepressant medication) willingly without a prescription when it was requested by its brand name (Prozac, Indianapolis, Indiana, USA).^5^ Moreover, a recent Saudi study conducted among university medical students in Riyadh, Kingdom of Saudi Arabia revealed that 120 out of 726 students used sedatives and stimulants simultaneously.^6^

Pharmacists who allow self-prescription of these medications are failing to uphold professional standards; such unprofessional behavior can cause harm to patients. For instance, road traffic accidents,^7^,^8^ abuse,^9^,^10^ falls^11^ and cognitive impairment^12^ are possible outcomes of psychotropic medications if these medications are inappropriately used by a patient who self-prescribes. In 2007, the Food and Drug Administration updated a black box warning on all antidepressants to include warnings on increased risks of suicidal thinking and behavior in patients younger than 25 years during initial treatment stage.^13^ Hence, these medications must be used under the supervision of a physician. Furthermore, psychotropic medications place those in older age groups at a greater risk of developing side effects because of the physiological changes associated with aging.^14^–^16^ In general, a lack of trust in medical services, the high cost of health services, the low severity of symptoms, and previous experiences with the medication are commonly reported reasons for using medications without medical supervision.^3^,^17^ The aim of this study was to identify the reasons why adults seek psychotropic medications without a prescription in Riyadh, Kingdom of Saudi Arabia.

## Methods

### The expert study design

This observational, quantitative and cross-sectional study was carried out from November 2014 to August 2015. The study was given ethical approval by the Ethical Review Committee at King Saud University Medical City, Riyadh, Kingdom of Saudi Arabia. To obtain the consent of the participants, a detailed explanation of the aims of the study was included at the beginning of the questionnaire.

### Population

The study included Arabic speakers who are ≥18years old, who have obtained at least one psychotropic medication without a prescription from retail pharmacies in the past 4 weeks and who live in Riyadh, the capital of Saudi Arabia. Participants who did not meet the study inclusion criteria, who used psychotropic medications with a prescription or who used non-psychotropic medications were excluded. The sample size was calculated using the standard equation (n=Za2p(1-p)/d2, Za=1.96, p=50%, D=5%, =384).

### Recruitment

Originally, retail pharmacies from the major areas of Riyadh were chosen using stratified random sampling techniques. However, when piloting the study, almost all retail pharmacy workers approached, refused to collaborate due to a fear of legal consequences. Then, we began collecting a convenience sample electronically plus distributing paper questionnaires among visitors of some retail pharmacies, as well as government and private psychiatric clinics, who obtained psychotropic medications without a prescription from retail pharmacies in Riyadh, Kingdom of Saudi Arabia.

### Data collection

Potential participants who visited retail pharmacies and psychiatric centers in different regions of Riyadh were asked to complete a self-administered questionnaire. An online web based version of the survey was also distributed among internet users via e-mail and social media (Twitter, WhatsApp, Facebook, web forums, etc.). The questionnaire consisted of 8 sections. The first section contained questions regarding demographic data; including age, gender, nationality, educational level, marital status and occupational status. The second section consisted of questions regarding the participant health information, including health insurance, and diagnosed chronic medical and psychiatric illness. The third part of the questionnaire contained questions related to psychotropic medications used by the respondents without a prescription. Participants were provided with a list of 13 most frequently dispensed psychotropic medications, with brand name examples of each medication, from which to choose, plus one fill-in option (others) to allow the participant to add another medication not included on the list. The third section also contained a question regarding the duration of medication use.

In the fourth section, respondents were asked to choose the symptom(s) that they used psychotropic medication(s) to treat. The fifth part asked on the sources of information and the way in which patients obtained psychotropic medications without a prescription. The sixth section of the questionnaire focused on the reasons that drove participants to use psychotropic medications without a prescription instead of seeking the help of a heath professional. In the seventh part, the validated Patient Health Questionnaire 9 (PHQ9) was used to measure the prevalence of major depressive disorder. Patient Health Questionnaire 9 is a validated self-administered screening questionnaire with a cutoff score of 10 for detecting major depressive disorder with a sensitivity and specificity of 88% when scoring ≥10.^18^,^19^ The last section included the validated Generalized Anxiety Disorder 7-item (GAD-7) scale to screen for general anxiety disorders. A cutoff point of 10 with a sensitivity of 89% and a specificity of 82% was used to identify probable cases of general anxiety disorder.^20^

The study questionnaire was piloted with 20 Primary Care Unit visitors at King Khalid University Hospital to test the validity of the questionnaire. A meantime of 9 minutes needed for completion of the questionnaire was recorded. The content of the study questionnaire was validated by experts in psychiatry and pharmacy. The wording and suggested answers were modified for some questions based on the feedback from experts and the pilot sample.

### Statistical analysis

The data were analyzed using the Statistical Package for Social Sciences version 21 (SPSS Inc., Chicago, IL, USA). Frequencies and percentages were used to express the descriptive results. Chi-square statistical analysis was used to test for associations. The limit for statistically significant differences was *p*≤0.05.

## Results

Four hundred and 29 patients completed the questionnaire, and 127 participants of them were excluded because they did not fit the inclusion criteria. Forty-six of the 127 excluded participants were excluded because they were using over the counter medications, 40 participants were using non-psychotropic medications, 20 participants were using psychotropic medications with prescriptions and the remaining 21 participants were excluded because have incomplete questionnaires with missing data. Eventually, 302 participants, 95% of them were approached electronically, were included finally in the analysis and their demographic characteristics are presented in **Table 1**. One hundred and eighty-eight (62.3%) participants had no health insurance. Two hundred and 28 (75.5%) participants reported having no chronic medical diseases. When asked if they had been diagnosed with a psychiatric illness, 197 (65.2%) indicated that they had not been diagnosed with a psychiatric illness.

**Table 1:** Demographic characteristics of the study population. (N=302)

Variables	n	(%)
*Age*		
18-25	100	(33.1)
26-35	130	(43.0)
36-45	48	(15.9)
46-55	18	(6.0)
56-65	5	(1.7)
≥ 66	1	(0.3)
*Gender*		
Male	181	(59.9)
Female	121	(40.1)
*Nationality*		
Saudi	287	(95.0)
Non-Saudi	15	(5.0)
*Education level*		
Illiterate	2	(0.7)
Intermediate	9	(3.0)
Secondary	60	(19.9)
University	175	(57.9)
Higher Education	56	(18.5)
*Marital status*		
Single	153	(50.7)
Married	137	(45.4)
Divorced	12	(4.0)
Widower	0	(0)
*Occupational status*		
Student	85	(28.1)
Employee	149	(49.3)
Self-employed	13	(4.3)
Retired	4	(1.3)
Unemployed	51	(16.9)

During the 4 weeks preceding the study, escitalopram was the medication most commonly used by participants (31.8%), followed by fluoxetine (23.2%) and paroxetine (21.9%) (**Table 2**). Most of the participants (56.0%) had used medication for one year or less, whereas the remainder (38.7%) had used medication for more than one year. Respondents reported taking psychotropic medications without a prescription mainly to treat symptoms of “feeling sad or depressed” (57.6%), “general anxiety” (41.4%), “cannot enjoy life anymore” (37.1%), “difficulty sleeping” (34.1%) and “social phobia” (26.2%). Family and friends were the most common sources of information related to the medications chosen, as indicated by 111 respondents (36.8%). Previous medication use (32.8%), internet and social media (33.1%) and recommendation by a pharmacist (17.5%) were other sources of information. Only 0.7% of the respondents reported that advertisements were a factor in choosing to obtain psychotropic medications without a prescription.

**Table 2:** Frequency of psychotropic medications used as reported by participants during the last 4 weeks. (N=302)

Medications	n	(%)
Escitalopram	96	(31.8)
Fluoxetine	70	(23.2)
Paroxetine	66	(21.9)
Propranolol	53	(17.5)
Mirtazapine	37	(12.3)
Pregabalin	37	(12.3)
Clozapine	29	(9.6)
Quetiapine	28	(9.3)
Venlafaxine	21	(7.0)
Fluvoxamine	17	(5.6)
Clomipramine	14	(4.6)
Lamotrigine	11	(3.6)
Olanzapine	9	(3.0)
Others	64	(21.2)

Most of the participants (66.6%) obtained medication from pharmacists by asking for it by name, whereas 13.6% obtained medication by showing a medication package to a pharmacist, and 12.9% obtained medication by sharing with relatives or friends who used medication with a prescription. Thirty-three (10.9%) used an old prescription, and 7.0% described their symptoms to a pharmacist. Among the reasons given for taking psychotropic medications without a prescription, 128 (42.4%) of the respondents believed that their symptoms were not serious enough to require the help of a physician. The high cost of psychiatric clinics, Lack of trust in psychiatrists and a lack of time were other reported reasons, as indicated by 28.5% 22.5% and 22.5% of the respondents, respectively (**Table 3**).

**Table 3:** Reasons underlying use of psychotropic medications without a prescription (N=302).

Variables	n	(%)
Symptoms are not serious	128	(42.4)
High cost of psychiatric clinics	86	(28.5)
Lack of trust in psychiatrists	68	(22.5)
Lack of time	68	(22.5)
Unavailability of medical services near residence	58	(19.2)
Crowded psychiatric clinics	56	(18.5)
Previous visits to a psychiatrist were not helpful	56	(18.5)
Fear of community judgment regarding visiting a psychiatric clinic	56	(18.5)
Transportation difficulties	34	(11.3)
Others	20	(6.6)

The prevalence of depression among the participants was 46.0%, as determined using the Patient Health Questionnaire (PHQ-9). Most of the participants rated doing their work, taking care of things at home or getting along with other people, as “somewhat difficult”(43.4%) or “not difficult at all” (20.2%) because of depression. Furthermore, the Generalized Anxiety Disorder 7-item (GAD-7) scores showed that 41.7% participants had general anxiety disorder. 41.1% described their lives as being “somewhat difficult” because of anxiety, whereas 21.5% described their lives as being “not difficult at all “with respect to taking care of things at home or getting along with other people. Demographic data, health information, duration of medication use, PHQ9 scores and GAD7 scores were the variables tested for potential associations with the reasons underlying the use of psychotropic medications without a prescription (**Table 4**). Additionally, demographic data, health information and duration of use were tested for potential associations with PHQ9 and GAD7 scores (**Table 5**). Overall, participants who had psychiatric illnesses were more likely to attribute their use of psychotropic medications without a prescription to their feeling that previous visits to their psychiatrists were not helpful (*p*=0.001). Males reported more a lack of trust in psychiatrists as a reason for using psychotropic medications without a prescription (*p*=0.042) (**Table 4**).

**Table 4:** Analysis of reasons underlying use of psychotropic medications without a prescription and related factors in the study population. (N=302)

Variables	Previous visits were not helpful	Lack of time	My symptoms are not serious	Fear of community judgment	Unavailability of medical services	Transportation difficulties
	*P*-value					
Age	NS	NS	NS	NS	NS	NS
Gender	NS	0.029	NS	NS	NS	0.000
Education level	NS	NS	NS	NS	NS	NS
Marital status	0.046	0.021	NS	NS	NS	0.014
Occupational status	NS	NS	NS	0.034	NS	0.043
Health insurance	NS	NS	NS	NS	NS	NS
Chronic disease	NS	NS	NS	NS	NS	NS
Psychiatric illness	0.001	NS	0.000	NS	NS	NS
Time of usage	NS	0.038	NS	NS	NS	NS
PHQ score	NS	NS	NS	NS	NS	NS
GAD score	NS	NS	NS	NS	NS	NS

PHQ - Patient Health Questionnaire, GAD - Generalized Anxiety Disorder 7-item, NS - not statistically significant

**Table 5:** Analysis of PHQ-9 and GAD-7 scale results with demographic data of the study population, using psychotropic medications without a prescription. (N=302)

Variables	PHQ score	GAD score
	(<10 Versus ≥10)	
	*P*-value	
Age	NS	NS
Gender	NS	NS
Education level	NS	0.050
Marital status	NS	NS
Occupational status	NS	NS
Health insurance	NS	NS
Chronic disease	NS	NS
Psychiatric illness	NS	NS
Time of usage	NS	NS

PHQ-9 - the 9-item Patient Health Questionnaire, GAD-7- the 7-item Generalized Anxiety Disorder Scale, NS - Not statistically significant

## Discussion

In Saudi Arabia, the malpractice of pharmacists dispensing prescription-only medications without a prescription has been reported in the literature.^5^ This study revealed several significant factors influencing patients’ decisions to visit retail pharmacies for psychotropic medications.

Among psychotropic medications, escitalopram was ranked as the drug most commonly used without a prescription by majority of the participants. Escitalopram is a selective serotonin reuptake inhibitor (SSRI) approved for treating major depressive disorder in adults and adolescents 12 or older, as well as for treating generalized anxiety disorder in adults.^21^ Despite being relatively safe agents, SSRIs including Escitalopram has been associated with increased risk of treatment emergent suicidal gestures/events in patients younger than 25 years old,^13^ GI bleeding,^22^ stroke^23^ and serotonin syndrome.^24^ Hence, the use of escitalopram and other SSRIs must be monitored closely by a physician. The findings of this study also revealed that three atypical antipsychotics (quetiapine, olanzapine, and clozapine) were used by 21.9% of the participants. Second-generation antipsychotic (SGA) side effects include weight gain, diabetes, dyslipidemia;^25^,^26^ hyperprolactinemia;^27^ neuroleptic malignant syndrome;^28^ cardiac arrhythmias;^29^ and sudden cardiac death.^30^

Clozapine, in particular, was used by 9.6% of the study participants without a prescription. Although clozapine is uniquely efficacious in treating treatment-resistant schizophrenia, it requires monitoring of white blood cell counts (WBC) due to a higher risk of potentially life-threatening agranulocytosis(1%).^31^ Clozapine is also associated with a higher risk of seizure^32^ and myocarditis.^33^ Moreover, pregabalin was used by 12.4% of the study participants without a prescription. Recently, there has been growing concern on the addictiveness of pregabalin.^34^ Interestingly, none of the benzodiazepines in our study was ranked among the most commonly used psychotropic medications without a prescription, although, benzodiazepine abuse remains common problem worldwide.^35^,^36^ Benzodiazepines in Saudi Arabia are grouped under the category of controlled and Narcotics medication, therefore, they are strictly prescribed and available mostly in hospitals.^37^

Because there are insufficient data on the use of psychotropic medications without a prescription in Kingdom of Saudi Arabia or worldwide, it is challenging to make comparisons at a national or international level. Nevertheless, the prevalence and the pattern of illegal psychotropic medication usage in this study appear to be significant and should be taken seriously.

Five hundred and fifty-two medications were used by 302 participants for a mean of 1.83 medications per participant. The authors noted a 22.2% difference between the percentages of medications used by males and females, with 61.1% of medications being used by males and 38.9% by females. Participants who had not been diagnosed with a psychiatric disorder used 101 more medications than the participants who had been diagnosed with a psychiatric disorder. The most common sources of information that the respondents mentioned were family and friends. The influence of family and friends on health decision-making has been addressed in the literature.^3^,^38^,^39^ These results may be explained by the fact that family and friends are trusted and reliable and quick sources of information due to their previous experiences or knowledge regarding medication use. Moreover, some of the participants may have witnessed the relief of similar symptoms in a family member or friend who used the same medication.

In this study, it was found that the most common cause of seeking psychotropic medications without a prescription was that the participants did not consider their symptoms to be serious enough to seek the help of a psychiatrist. A possible reason for this may be low awareness on common psychiatric disorder symptoms. Compared with another study conducted in Iran in 2013 among pharmacy visitors, the most reported factors for self-medicating were previous medication use, symptom improvement, and similarity to prescribed medications.^40^

Our study showed that a high proportion of the study participants have a diagnosable psychiatric illnesses (46% likely had major depression based on PHQ-9 scale and 41.7% had generalized anxiety disorder based on GAD-7 scale). This result is in contrast to a smaller proportion of participants who self-reported having been diagnosed by psychiatrist in the past with any psychiatric illness (34.8%). In this study, the prevalence of depression was 46% and 42% for the anxiety. The rates of depression and anxiety were much higher than that reported in primary care setting in Saudi Arabia, which was 18.8% for depression^41^ and 5.3% for anxiety disorder.^42^

Additionally, our study found that 62.3% of study participants had no health insurance. In Saudi Arabia, all Saudis can seek medical/psychiatric help in government medical centers for free. However, the waiting list for appointments is very long. Moreover, those who carry health insurance can seek medical care in most private medical settings for most medical problems but cannot receive treatment for dental, plastic surgery or psychiatric care, as these services are not covered by basic health insurance.^43^ This differs from insurance medical care systems in most parts of the world and may partially explain why health insurance was not significantly associated with dispensing psychotropic medications without a prescription at retail pharmacies in Kingdom of Saudi Arabia. The poor accessibility to mental health care services, psychiatric stigma and other cultural factors could explain partly the high prevalence of psychiatric illnesses among faith healing users in Saudi Arabia especially among undereducated, poor income males.^44^ Thirty-nine percent of the study participants used medications without a prescription for more than 1 year, which could make them prone to drug dependence on medications with addiction potential like pregablin^10^ and increase the risk of withdrawal syndrome with medications like paroxetine and venlafaxine, which have been used by 29% of the study participants; especially if the discontinuation is abrupt.^45^ This study had several limitations. First, no similar studies were found for comparison. Second, the results of this study cannot be generalized to the rest of Riyadh city due to the use of a convenience sampling technique. Third, the sample size was small because of the social stigma associated with psychiatric conditions. Forth, using self-rated scales to diagnose depression and anxiety may be less accurate compared with diagnostic interviews conducted by a healthcare professional. Last, as this was a self-reported study, the possibility of recall bias cannot be excluded.

In conclusion, the primary reason underlying the use of psychotropic medications without a prescription that was reported by the participants was that they did not consider their symptoms to be serious enough to visit a health clinic or psychiatric center, followed by the high cost of psychiatric services. Escitalopram was the drug that was most commonly used without a prescription. Three atypical antipsychotics (quetiapine, olanzapine, and clozapine) were used by more than one-fifth of the study participants. Depression and anxiety were identified in the majority of participants obtaining psychotropic medications without a prescription at retail pharmacies.

We recommend implementing strong and effective rules and policies to prevent pharmacists in retail pharmacies from dispensing non-OTC medications in general and psychotropic medications in particular without a prescription. This study calls for improving access to mental health services. Additionally, awareness and educational programs demonstrating the harm of using psychotropic medications without a prescription and the symptoms of common psychiatric disorders should be implemented. Future research should explore other aspects of this phenomena and interview participants to ensure more reliable results.
